# A Facile One-Pot Approach to the Fabrication of Nanocellulose–Titanium Dioxide Nanocomposites with Promising Photocatalytic and Antimicrobial Activity

**DOI:** 10.3390/ma15165789

**Published:** 2022-08-22

**Authors:** Roberta G. Toro, Abeer M. Adel, Tilde de Caro, Bruno Brunetti, Mona T. Al-Shemy, Daniela Caschera

**Affiliations:** 1 Institute for the Study of Nanostructured Materials, National Research Council, Via Salaria Km 29,300, Monterotondo Stazione, 00015 Rome, Italy; 2 National Research Centre, Cellulose and Paper Department, 33El-Bohouth St. (Former El-Tahrir St.), Dokki, Giza P.O. Box 12622, Egypt; 3 Dipartimento di Chimica, Università degli Studi di Roma “La Sapienza” Institute for the Study of Nanostructured Materials, National Research Council c/o, Piazzale Aldo Moro 5, 00185 Rome, Italy

**Keywords:** nanotechnology, titanium dioxide, nanocellulose, hybrid nanocomposite, photocatalytic activity, photodegradation kinetic, antimicrobial effects

## Abstract

The combination of cellulosic materials and metal oxide semiconductors can provide composites with superior functional properties compared to cellulose. By using nanocellulose derived from agricultural waste, we propose a one-pot and environmentally friendly approach to the synthesis of nanocellulose–TiO_2_ (NC–TiO_2_) nanocomposites with peculiar photocatalytic activity and antibacterial effects. The as-prepared NC–TiO_2_ composites were fully characterized by different techniques, such as X-ray diffraction (XRD), μ-Raman, Fourier transform infrared spectroscopy (FTIR), thermogravimetry analysis (TGA), scanning electron microscopy (SEM), transmission electron microscopy (TEM), and diffuse reflectance spectroscopy (DRS). The results showed that well crystalline anatase TiO_2_ nanoparticles of about 5–6 nm were obtained. The photocatalytic activity in particular was evaluated by using methyl orange (MO) solution as a target pollutant at different pH values. It was found that all the tested NC–TiO_2_ nanocomposites showed stable photocatalytic activity, even after consecutive photocatalytic runs. In addition, NCT nanocomposites with higher TiO_2_ content showed degradation efficiency of almost 99% towards MO after 180 min of UV illumination. Finally, NC–TiO_2_ nanocomposites also showed intriguing antimicrobial properties, demonstrating to be effective against Gram-positive (*Staphylococcus aureus*, *Bacillus subtilis*) with 20–25 mm of inhibition zone and Gram-negative bacteria (*Escherichia coli*, *Pseudomonas aeuroginosa*) with 21–24 mm of inhibition zone, and fungi (*Candida albicans*) with 9–10 mm of inhibition zone.

## 1. Introduction

Hybrid organic–inorganic materials have been the focus of intense research in the last few decades for the numerous advantages they potentially offer in many technological fields, such as optics, electronics, ionics, mechanics, and biology [1]. The particular interest around this class of materials relies on the enhanced optical, mechanical, electrical, and thermal properties the hybrid composites show with respect to the organic and inorganic counterparts. The new properties, in most cases, can be further tuned by simply varying the precursors ratio, their chemical functionalities, and, hence, the mutual chemical and/or physical interactions between the inorganic and organic components [2,3].

In this context, cellulose, the most abundant biopolymer in nature, is considered an excellent natural material with remarkable advantages in terms of good biodegradability, biocompatibility, and low cost, and can represent an ideal platform for the design and fabrication of hybrid organic–inorganic nanocomposites. Cellulose can be obtained from a variety of biomass sources, including wood, cotton, linen, bamboo, and agriculture straw plant cells [4]. The repeat unit of cellulose consists of a cyclic backbone of sugar rings with three hydroxyls on each ring. The presence of such high-density oxygenated functional groups as hydroxyl and ether groups provides peculiar chemical functionalities and makes nanocellulose an ideal matrix to promote the synthesis of metallic and metal oxide nanoparticles [5,6]. Many studies have indicated that nanocellulose can act either as templates or precursors [7,8]. Luo et al. [9], for example, reported the hydrothermal synthesis of hierarchical fibrous anatase–cellulose composites and demonstrated that, although no additional functional groups or binders were introduced into the cellulose, the anatase nanoparticles were homogeneously anchored to each cellulose nanofiber. Caschera et al. [10] fabricated silver–carboxylate nanocellulose (Ag–ONCs) nanocomposites by a facile two-step green method where the ONCs acted as a template and reducing agent for silver nanoparticles formation due to the specific hydroxyl and carboxyl groups on the cellulose surface. Recently, we demonstrated that paper sheets fabricated from nanocellulose recycled by agriculture wastes could be used as stable and robust matrices for the ex situ loading of pre-formed TiO_2_ nanoparticles [11] since the hydroxyl groups on the surface of nanocellulose fibers offered stable anchoring sites for the stabilization of the TiO_2_ nanoparticles. The TiO_2_-modified paper sheets showed not only new photocatalytic activity towards methylene blue but also improved mechanical performances in terms of better tensile index, breaking length, tear and burst index, and air permeability. Methyl orange (MO) is an azo dye commonly known as a pH indicator. It is very soluble in aqueous solution, and it is extensively used in several industries, including the textile, paper, printing, and food industries, and mostly discharged in industrial wastewater [12]. Furthermore, MO is toxic and brings about serious water pollution, causing anomalous coloration to the surface water, blocking activity of photosynthetic bacteria and aquatic plants, and further representing a hazard to all mankind [13]. In this context, the removal of MO from effluent is strongly required.

TiO_2_ is certainly the most studied inorganic semiconductor as it is the key component in many relevant applications, including aspects related to the environment and clean energy, antimicrobial agent, food, and biomedical [14]. In particular, as a photocatalyst, TiO_2_ has many advantages in terms of strong oxidation activity towards a wide range of organic pollutants, environmental stability, easy synthesis, and low cost [15,16,17,18,19]. However, its practical application as a photocatalyst is still limited due to some issues regarding stability under real operating conditions (e.g., spontaneous aggregation of TiO_2_ nanoparticles under the complex physical and chemical wastewater conditions), recovery, and reuse of the catalyst. In this perspective, the development of TiO_2_ bio-composite photocatalysts can be an effective approach to overcome these limits as it can promote a more efficacious dispersion of catalyst nanoparticles and enhance the functional properties of the as-synthesized composite. Liu et al. [20] reported the preparation of anatase TiO_2_/cellulose composites at low temperature, and the prepared composite displayed outstanding photocatalytic performance for the degradation of methyl orange. However, they used highly corrosive nitric acid to catalyze the hydrolysis of titanium precursor, and, in addition, the final stage of lyophilization was time-consuming and required quite expensive equipment. Xiao et al. [21] instead synthesized TiO_2_ nanoparticles by using nanofibrillated cellulose as a template and the hazardous TiCl_4_ as a titanium source. The cellulose was then removed by the final annealing step carried out at different temperatures in order to obtain crystalline TiO_2_ nanoparticles.

In this study, we propose the one-pot synthesis of hybrid nanocellulose–TiO_2_ nanocomposites (NC–TiO_2_) for the decolorization of MO under UV light. The NC–TiO_2_ nanocomposites were obtained by a simple, cost effective, and environmentally friendly sol–gel method using non-toxic chemicals (water and acetic acid) and nanocellulose (NC) extracted from agriculture residues [22]. The NC–TiO_2_ nanocomposites with different amounts of NC were prepared at room temperature using a green solvent, such as distilled water, and mild acetic acid as a catalyst for the hydrolysis of the titanium butoxide precursor. A cost-effective aging process at room temperature and ambient pressure was carried out instead of traditional heat treatment to favor the complete crystallization of TiO_2_. Furthermore, the nanocellulose (NC) obtained by recycling agricultural waste in the present study had high crystallinity and active carboxylate groups, in addition to the intrinsic hydroxyl groups of cellulose, which are expected to stabilize TiO_2_ nanoparticles during the hydrolysis step of the titanium precursor. A complete physicochemical characterization of the NC–TiO_2_ composites was carried out using X-ray diffraction (XRD), attenuated total reflectance-Fourier transform infrared (ATR-FTIR), μ-Raman spectroscopy, UV–visible spectroscopy, field emission scanning electron microscopy (FESEM), and thermogravimetric analysis (TGA). The NC–TiO_2_ nanocomposites were tested in the photodegradation of the reactive azo dye methyl orange at different pHs, and their reusability in the degradation process was evaluated as well. Furthermore, the antibacterial properties were also investigated by monitoring the inhibition growth of NCT nanocomposites towards Gram-positive and Gram-negative bacteria and fungi.

## 2. Materials and Methods

### 2.1. Materials and Reagents

The chemicals used for sol−gel synthesis include titanium (IV)-n-butoxide (≥99% Alfa Aesar, Harverhill, MA, USA) and glacial acetic acid (Alfa Aesar, Harverhill, MA, USA). The bagasse fibers were obtained from the Egyptian pulp and paper industry (Quena Company of Cairo, Egypt). Methyl orange was purchased at Sigma-Aldrich (St. Louis, MO, USA). Deionized water was used in all the experiments.

### 2.2. Synthesis of NC–TiO_2_ Nanocomposite

Nanocellulose (NC) was prepared from bagasse raw material using ammonium persulphate hydrolysis (APS) method as previously reported [10,23]. Briefly, 1 g of raw bagasse fibers is hydrolyzed with 1.5 M APS solution at liquor ratio 1:100 and 60 °C for 24 h under continuous stirring. The resulting white suspension is dialyzed against distilled water till the solution reached pH ≈ 5. NC with pH 7 is prepared by adding 1 M NaOH.

The NC–TiO_2_ nanocomposites were prepared by a simple and low-cost sol–gel method. Titanium (IV)-n-butoxide (Ti(OBut)_4_)was used as Ti precursor. First, the proper amount of NC was dispersed in 100 mL of distilled water and then sonicated in an ice bath at 0 °C for 10 min to promote high dispersion. Ti(OBut)_4_ and glacial acetic acid were carefully mixed in a fixed volume ratio of 1:2, under vigorous stirring at room temperature. After 10 min, the acidic dispersion containing the titanium precursor was added dropwise to the prepared NC dispersion under constant and vigorous stirring. Different amounts of NC were used to prepare the aqueous dispersion, and the resulting NC–TiO_2_ nanocomposites were denoted by their NC content ranging from 10 to 80 wt% with respect to TiO_2_. The mixture was vigorously stirred at room temperature for 24 h, and then the resulted aqueous white dispersion was stored in dark conditions without stirring at room temperature and atmospheric pressure for five days. The NC–TiO_2_ powders were precipitated by adding a 10% *w*/*w* sodium carbonate solution until a neutral pH was reached and then collected by centrifugation at 10,000 rpm (Thermo Scientific IEC CL31R Multispeed centrifuge, Waltham, MA, USA), washed three times with deionized water, and finally dried at 45 °C under vacuum. The final product exhibited a light-yellow color, which was indicative of the formation of the NC–TiO_2_ nanocomposite. The same procedure without nanocellulose was applied for the synthesis of pure TiO_2_. The NC–TiO_2_ nanocomposites with different amount of NC were coded as NC10–TiO_2_, NC20–TiO_2_, and NC80–TiO_2_ depending on the % wt of NC.

### 2.3. Characterization of NC–TiO_2_ Nanocomposite

Structural characterization was performed by *X-ray Diffraction (XRD)* using a Siemen D5000 X-ray diffractometer, equipped with a CuKα (λ = 1.5406 Å) radiation operating at 40 kV and 30 nA. Raman spectra were collected with a *µ-Raman spectrometer* (Renishaw RM 2000, Gloucestershire, UK) operating in the backscattering configuration, and equipped with a Peltier cooled charge-coupled device (CCD) camera in combination with a Leica optical microscope with a 50× objective. Measurements were performed using the 785 nm emission line of a laser diode. *Fourier transform infrared (FTIR) spectroscopy* measurements were carried out using an Alpha FT-IR spectrometer (Brucker Optics, Ettingen, Germany) equipped with an external reflection exchangeable sampling module. The spectra were collected as the average of at least 200 scans at a resolution of 4 cm^−1^ in the frequency range 4000–500 cm^−1^. *Thermogravimetric analysis (TGA)* was performed on Ugine-Eyraud Model B60 Setaram thermobalance under static air, at constant heating rate of 5 °C/min, using a platinum crucible. UV−vis diffuse reflectance spectra were recorded using a Jasco double-beam V660 UV−vis spectrophotometer (Tokyo, Japan) in reflectance mode, equipped with a 60 mm integrating sphere and BaSO_4_ as standard diffuse reflectance material. Surface morphology was investigated by *SEM TESCAN VEGA 3* equipped with a LaB_6_ filament. *Transmission Electron Microscope (TEM)* analysis was carried out using a high-resolution JEOL-JEM 2100 (Tokyo, Japan).

### 2.4. Photocatalytic Activity of NC–TiO_2_ Nanocomposite

Photocatalytic degradation experiments were carried out at room temperature using MO as a model dye. 10 mg of NC–TiO_2_ nanocomposites was added to a beaker containing 50 mL of 25 mg/L MO aqueous solution at different pH (pH = 2, 7, 10). The pH of solution was adjusted by adding HCl or NaOH. A 39 W lamp (λ = 365 nm) was used as the light source. Before irradiation, the samples were kept in the dark for 1 h to ensure the adsorption–desorption equilibrium of MO on the surface of the nanocomposites. The beaker was set at a distance of 9 cm from the light source and irradiated under UV light. The absorbance was taken at given time intervals and the degradation rate was calculated by using the following equation, *Degradation rate (%)* = $\left(C_{0}-C_{t}\right)/C_{0}\;\times 100$, where C_0_ is the initial concentration and C_t_ is the concentration of MO at time t. For comparison, an extra test was carried out in the presence of pure TiO_2_ under the same experimental conditions.

### 2.5. Antimicrobial Activity of NC–TiO_2_ Nanocomposites

The antimicrobial examinations of prepared NC–TiO_2_ nanocomposites were carried out by using qualitative process. The qualitative test is accomplished through solid media (LB agar) using the agar diffusion disk method (AATCC Test Method 147-1988). The qualitative antimicrobial activity of the NC–TiO_2_ nanocomposites was examined on four different bacterial species G(+) bacteria as *Staphylococcus aureus, Bacillus subtilis*; G(−) bacteria as *Escherichia coli*, *Pseudomonas aeuroginosa*, and a fungi *(Candida albicans).* Blank paper disks (Schleicher & Schuell, Spain) with a diameter of 10 mm are soaked by 10 µL of the stock NC–TiO_2_ solutions (nominal NC–TiO_2 conc_ = 0.3 mg/mL) and put on LB Agar previously treated with the microorganism. After 48 h of incubation, the diameters of the resulted growth inhibition zones were measured and the averaged and mean values recorded in millimeters were tabulated. The antimicrobial agents Ampicillin and Amphotericin B were used as standard reference for G(+) & G(−) bacteria and fungi, respectively. The activity index (%) was calculated as [24]:

Activity index (%) = 100× [zone of inhibition by tested sample (mm)/zone of inhibition by standard (mm)].

## 3. Results

### 3.1. Structural and Morphological Characterization of NC–TiO_2_ Nanocomposites

**Figure 1:**
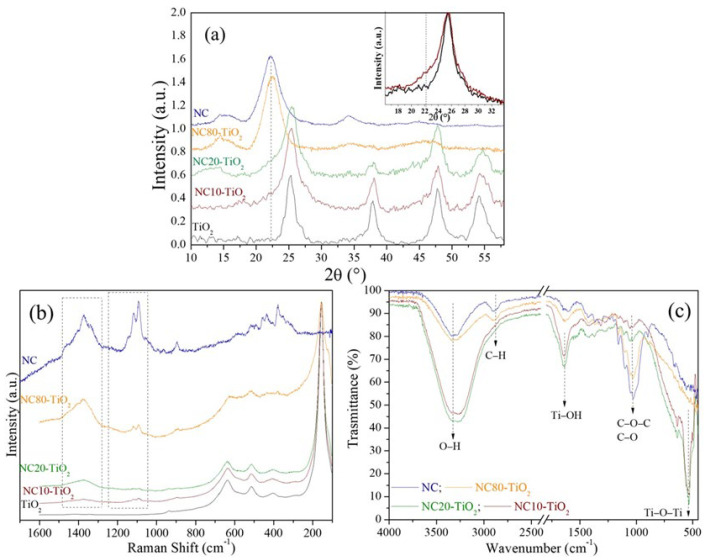
(**a**) XRD patterns of (**–**) NC, (**–**) TiO_2_, (**–**) NC80-TiO_2,_ (**–**) NC20-TiO_2_, and (**–**) NC10–TiO_2_ nanocomposites. The inset shows a comparison between the (101) peak of TiO_2_ and NC20–TiO_2_; (**b**) µ- Raman spectra and (**c**) FTIR spectra for nanocellulose (NC) and NC–TiO_2_ nanocomposites.

The X-ray powder diffraction (XRD) analysis performed on the NC–TiO_2_ nanocomposites pointed to the formation of TiO_2_ in the anatase crystalline phase. In Figure 1a, the XRD patterns of the NC–TiO_2_ nanocomposites are shown; the corresponding patterns of NC and neat TiO_2_ were also provided for comparison. The XRD patterns of the NC–TiO_2_ nanocomposites showed diffraction peaks at 2θ = 25.23°, 37.71°, 47.72°, 54.16°, and 55.32°, which correspond to the tetragonal crystal planes (101), (004), (200), (105), and (211), respectively, of the anatase TiO_2_ (JCPDS file 21-1272). The crystalline peaks characteristic of NC can hardly be seen, probably because of the low amount of NC in the nanocomposite. However, a closer look at the XRD patterns of the NC–TiO_2_ samples revealed the presence of a shoulder at lower 2θ values for the (101) diffraction peak (inset Figure 1) due to the contribution of the (200) peak of the nanocellulose. The broad XRD peaks further suggested that the NC–TiO_2_ nanocomposites had nanometric sizes. The crystalline sizes were determined using the Debye–Scherrer equation, D = Kλ/βcosθ, where *D* is the crystal size, *K* is dimensionless constant (0.9), *λ* is the wavelength of X-ray, *β* is the full width at half-maximum (FWHM) of the diffraction peak, and *θ* is the diffraction angle. The average crystalline sizes (D) of NC–TiO_2_ nanocomposites were found to be very similar, 5.73 and 5.81 nm for NC10–TiO_2_ and NC20–TiO_2_, respectively. There was no obvious difference in the particle size of the TiO_2_ nanoparticles with the nanocellulose as the template. Moreover, the calculated D values were less than the size of pure TiO_2_ (D_TiO2_ = 7.10 nm). Biomolecules, such as cellulose, can control the particle size of the TiO_2_, acting as a limiting spacer, and, therefore, the TiO_2_ crystal growth was limited by interactions between NC and TiO_2_ [25]. In the perspective of photocatalytic applications, small crystals can provide more available active sites that absorb more photon and produce more electrons-holes, thus enhancing the photocatalytic activity of the system.

The NC–TiO_2_ nanocomposites were further investigated by Raman spectroscopy, and the results were reported in Figure 1b. The Raman spectrum of neat NC confirmed the presence of peaks related to cellulose I structure [26]. In particular, the spectrum reported at 1093 and 1117 cm^−1^ the bands characteristic of the β-1,4-glucosidic linkages of the glucopyranose units of cellulose, the bands related to H-C-H and to H-O-C bending in the 1300–1500 cm^−1^, and, at 329, 378, and 435 cm^−1^, the bands related to the cellulosic ring deformation [27]. These bands were also visible in the Raman spectra of NC–TiO_2_ nanocomposites in addition to the high intensity bands at 155, 400, 514, and 638 cm^−1^, typical of TiO_2_ anatase, thus confirming that anatase was the predominant phase. No rutile or brookite phase bands were detected in the Raman spectra, as already evidenced by XRD analyses.

The chemical nature of surface functional groups in NC–TiO_2_ nanocomposites was further investigated by FTIR spectroscopy (Figure 1c). The FTIR spectra of nanocomposites showed an intense absorption below 800 cm^−1^, which derived from the contribution of the Ti–O bond in TiO_2_. In addition, the characteristic peaks of cellulose were also visible: the broad band in the 3600–3000 cm^−1^ region due to stretching vibrations of intra and inter O–H bonds, and the large absorptions in the 1200–860 cm^−1^ spectral region assigned to the glucosidic ring vibrations of C−H, C−O, and C−O−C [28,29]. These NC–TiO_2_ bands represented a unique fingerprint of cellulose, and their presence, although less intense, in the spectra of the NC–TiO_2_ nanocomposites, suggested that the cellulose retained its chemical structure. Nevertheless, the FTIR of the NC–TiO_2_ nanocomposites showed intense absorption peaks associated with the O–H stretching at 3300 cm^−1^ and the O–H bending at 1636 cm^−1^, which were shifted to lower wavenumbers with respect to those observed in nanocellulose. The observed shift was correlated to the involvement of –OH groups in the formation of a strong intermolecular hydrogen bonding interaction between the hydroxyl groups in the cellulose chain and Ti–OH groups on the surface of TiO_2_ nanoparticles [30,31]. Furthermore, the increase in the –OH surface groups is particularly interesting because it may affect positively the NC–TiO_2_ nanocomposites’ photodegradation activity as they can promote the formation of ^•^OH radicals during the photocatalytic process [32].

**Figure 2:**
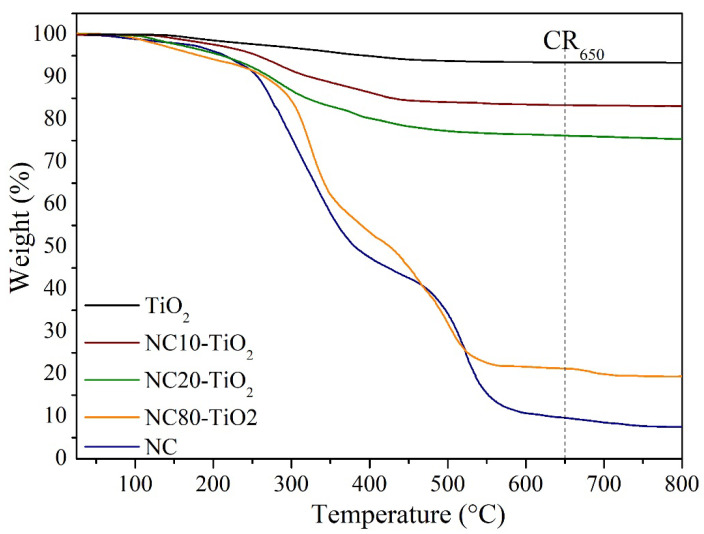
TGA of (**–**) TiO_2_, (**–**) NC, (**–**) NC10-TiO_2_, (**–**) NC20-TiO_2_, and (**–**) NC80-TiO_2_.

Thermogravimetric analysis (Figure 2) was carried out to obtain information on the thermal stability of the synthesized NC–TiO_2_ nanocomposites. TG curves of TiO_2_ and NC were also reported for comparison. The NC–TiO_2_ nanocomposites decomposed at a relatively higher temperature than NC (Figure 2). As expected, the TG curve of TiO_2_ showed a small weight loss of about 2% in the range 105–250 °C, related to the total content of residual water, acetic acid, and some organic groups deriving from the synthetic procedure. In the case of the NC10–TiO_2_, the TG curve showed an initial weight loss of about 5% up to 130 °C caused by the removal of physically absorbed water, followed by a second weight loss step of 16.8% in the 130–520 °C range, attributed to the burning of the carbon species in the nanocomposites. Further, there was no significant weight loss observed above 520 °C, and an RC_650_ of about 84% remained. A similar trend was observed also for the NC20–TiO_2_ except that for the lower RC_650_ of about 76% at 650 °C. Contrarily, the NC80–TiO_2_ followed a thermal degradation path similar to that of the NC, with two distinct steps of decomposition. The first step between 215° and 400 °C with a weight loss of 39% could be attributed to the reactions of dehydration and decarboxylation of the NC that produced the combustion gases, such as levoglucosan [33]. The second step in the range of 400–580 °C with RC_650_ ≈ 21% was the oxidative decomposition of the char formed from the first step [23,34]. In all cases, the residue was the inorganic component of TiO_2_, so we can infer the TiO_2_ content was about 84, 76, and 21 by wt. % for NC10–TiO_2_, NC20–TiO_2_, and NC80–TiO_2_, respectively.

Assuming pseudo-first-order kinetics, the kinetic parameters of each step in the pyrolysis range were estimated by employing Coats and Redfern’s theory [35]. The kinetic and thermodynamic parameters, such as activation energies (Ea; kJ mol^−1^), pre-exponential factors (A; s^−1^), enthalpy (∆H; kJ mol^−1^), entropy (∆S; JK^−1^ mol^−1^), and Gibbs free energy of activation (∆Ga; kJ mol^−1^), based on the weight loss data obtained from TG analyses, are given in Table S1. The Ea and A values were lower for NC10–TiO_2_ and NC20–TiO_2_ compared to NC80–TiO_2_, and this may be attributed to the presence of a higher content of non-flammable TiO_2_ covering NC nanocrystals, which requires lower energies to activate the pyrolysis reactions [36]. This means that the presence of TiO_2_ bonded to NC affected the thermal reaction pathway responsible for the different activation energies. From the overall results obtained in Table S1, we can observe the positive effect of TiO_2_ on the thermal stability of the corresponding NC–TiO_2_ nanocomposites, which follows the order NC10–TiO_2_ > NC20–TiO_2_ > NC80–TiO_2_.

**Figure 3:**
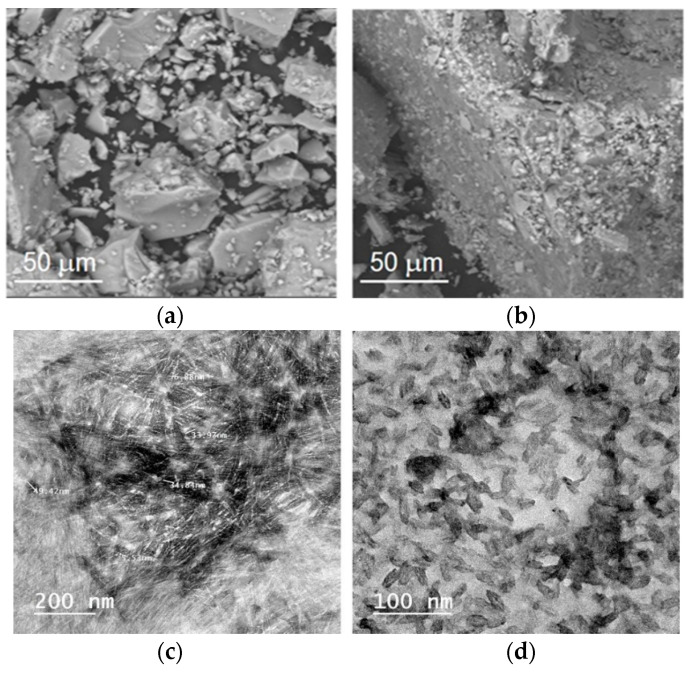
FE-SEM images of (**a**) TiO_2_, (**b**) NC20–TiO_2_, and TEM images of (**c**) NC and (**d**) NC20–TiO_2_.

The morphology of pure TiO_2_ and NC–TiO_2_ was studied by SEM, and the corresponding images were reported in Figure 3a,b. TiO_2_ is composed of large sheet-like aggregates, heterogeneous in size (Figure 3a). The NC–TiO_2_ composites had very similar morphologies as a result of the aggregation of several thousands of nanoparticles whose final size decreased slightly upon increasing the amount of nanocellulose (Figure 3b). The TEM image (Figure 3c) showed that the NC had nanosized rod-shaped whiskers, bunched together into a tendon-like structure, with an average NC width and length of about 6.2 ± 0.96 nm and 39.4 ± 8.68 nm, respectively. The NC–TiO_2_ nanocomposites had instead a granular shape (Figure 3d), with the TiO_2_ covering the surface of the NC. Moreover, the TiO_2_ particles not only covered the surface but also deposited on the pores of NC.

### 3.2. Photocatalytic and Antimicrobial Activity of NC–TiO_2_ Nanocomposites

**Figure 4:**
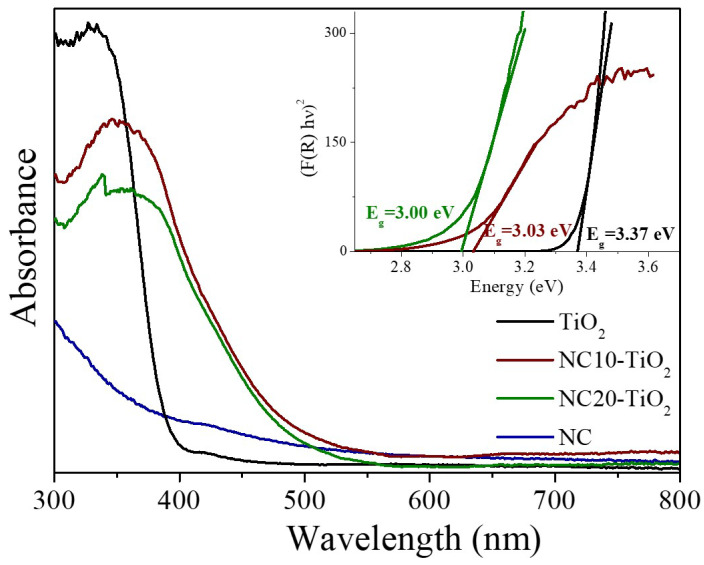
UV–vis spectra for (**–**) TiO_2_, (**–**) NC10–TiO_2_, (**–**) NC20–TiO_2_ nanocomposites, and (**–**) NC. The inset shows the Kubelka–Munk plot for (**–**) TiO_2_, (**–**) NC10–TiO_2_, and (**–**) NC20–TiO_2_ nanocomposites.

The optical properties of NC–TiO_2_ nanocomposites were investigated by UV–vis diffuse reflectance spectroscopy (Figure 4). The UV absorption spectra of the NC–TiO_2_ nanocomposites resembled that of pure TiO_2_, with the maximum UV absorption redshifted from 330 nm to 360 nm. The observed shift for the nanocomposites could be indicative of the chemical interactions of TiO_2_ NPs with the large molecular framework of the nanocellulose. The NCT nanocomposites also showed a certain level of visible light absorption, which, in general, could be associated with a decrease in the band gap following the formation of the NC–TiO_2_ hybrid nanocomposites [13,37]. The corresponding band gap values were obtained by plotting the Kubelka–Munk function against the photon energy (inset in Figure 4). Pure TiO_2_ showed a band gap of 3.37 eV, very close to the reported value for bulk TiO_2_ (3.40 eV). The band gap values of NCT nanocomposites were very similar and sensibly lower than that found for the pureTiO_2_ (E_g_ = 3.00 and E_g_ = 3.03 eV for NC10–TiO_2_ and NC20–TiO_2_, respectively). This result confirmed the bonding interactions between NC and TiO_2_ after the formation of the NC–TiO_2_ nanocomposites, and they were directly correlated to the observed absorption of visible radiation.

**Figure 5:**
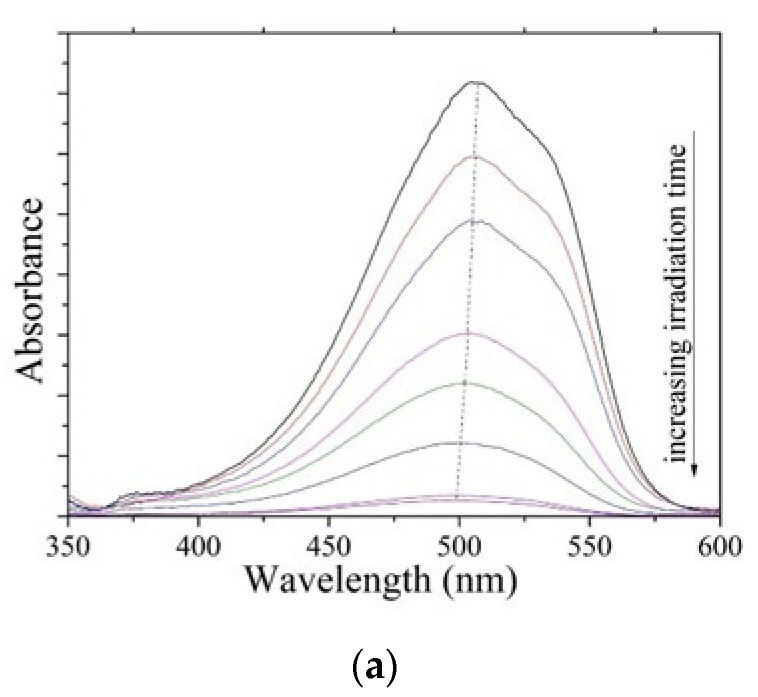

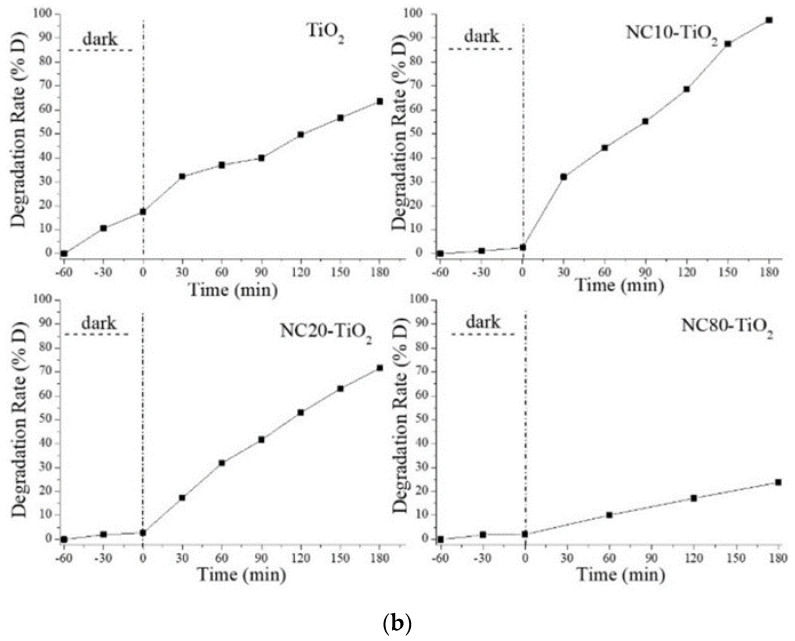
(**a**) Time–dependent UV–vis spectra of methyl orange and its degradation by NC10–TiO_2_ under UV irradiation; (**b**) quantitative evaluation of photocatalytic degradation rate of methyl orange by TiO_2_, NC10–TiO_2_, NC20–TiO_2_, and NC80–TiO_2_ under UV illumination.

Photodegradation tests were carried out in the presence of NC–TiO_2_ nanocomposite powders dispersed in 50 mL of aqueous solution with an initial MO concentration of 25 ppm at pH = 2. The pH of the solution was adjusted before irradiation, and it was not controlled during the course of the reaction. The residual concentration of the organic dye was followed by UV–vis spectroscopy monitoring the variation of absorbance at its λ_max_. Figure 5a showed the evolution of MO absorbance during the photocatalytic experiment. The MO absorption spectrum had a maximum at λ = 507 nm corresponding to the π→π* transitions of the dimethylamino electron donors. A progressive decrease in the π→π* MO absorption peak with the increasing irradiation time and a corresponding blue-shift during the kinetic experiments were observed, which confirmed the degradation of the dye associated with the removal of the N-methyl groups [38].

Figure 5b showed the degradation rates of MO by NC–TiO_2_ nanocomposites under UV light. Before UV irradiation, solutions containing the nanocomposites were stirred for 60 min in the dark at room temperature to establish the adsorption–desorption equilibrium. Apart from pure TiO_2,_ none of the nanocomposites showed noticeable absorption phenomena. During the first stage, indeed, it was observed that 17.5%, 2%, 2.5%, and 1.3% MO were absorbed for pure TiO_2_, NC10–TiO_2_, NC20–TiO_2_, and NC80–TiO_2_, respectively, indicating that the absorption had negligible effects on the removal of MO in the case of the NCT composites.

Under UV light and in the presence of the raw NC alone, MO did not display any significant photodegradation after 120 min, indicating that nanocellulose had no photocatalytic activity (data not shown). When NC–TiO_2_ samples were irradiated with UV light, the situation changed drastically. The highest degradation rate was reached by the NC10–TiO_2_, which degraded almost 99% of MO after 180 min of UV illumination, while NC20–TiO_2_ reached 72%. For NC80–TiO_2_, the photocatalytic activity was estimated at about 23% after 180 min. These findings confirmed that NC10–TiO_2_ composite showed excellent photocatalytic performance, similar and, in some cases, higher with respect to analogous systems, as reported in Table 1.

**Figure 6:**
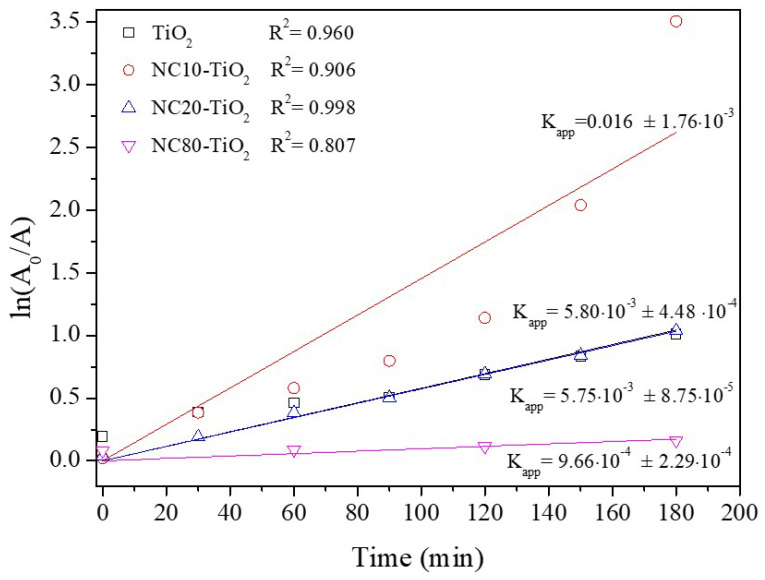
Kinetics of methyl orange photodegradation using NC–TiO_2_ nanocomposites.

The kinetics of the MO photodegradation was described by using the pseudo-first-order model ln(C_0_/C) = *k*_app_*t*, where C_0_ is the initial concentration of MO and *k*_app_ is the apparent rate constant. The kinetic and regression constants are reported in Figure 6. The NC10–TiO_2_ had the highest reaction rate constant (*k*_app_ = 0.016 min^−1^), about four times higher than that of bare TiO_2_, thus confirming the best degradation efficiency, suggesting that low amounts of nanocellulose had beneficial effects on the degradation efficiency of the corresponding NC–TiO_2_ nanocomposites. This result was in contrast with those reported in some cases, as in Ref. [39], according to which the efficiency in mefenamic acid (MEF) degradation decreased by increasing the TiO_2_ content in the NC–TiO_2_ nanocomposites. These differences suggest that not only the amount of NC in the nanocomposites affected the photodegradation activity but also the NC–TiO_2_ intrinsic chemical structure. In the present study, the observed behavior could be explained by considering the intrinsically different amounts of hydroxyl groups available at the surface of the catalyst particles. As already known, the species responsible for photodegradation of dyes in water were hydroxyl radicals, ^•^OH, and anion radical superoxides, $O_{2}^{-}$^•^. Hydroxyl radicals were produced by the photogenerated holes (h+) in the conduction band from the direct oxidation of H_2_O, OH^−^ ions, or terminal –OH groups on the surface of the catalyst. In this respect, NC–TiO_2_ nanocomposites with low amounts of NC provided a large number of hydroxyl groups on the catalyst surface, as evidenced by the FTIR results, which could promptly react with h^+^, locally producing ^•^OH radicals, which degraded the organic dyes in solution and reduced the electron-hole recombination responsible for the decrease in photocatalytic activity.

**Figure 7:**
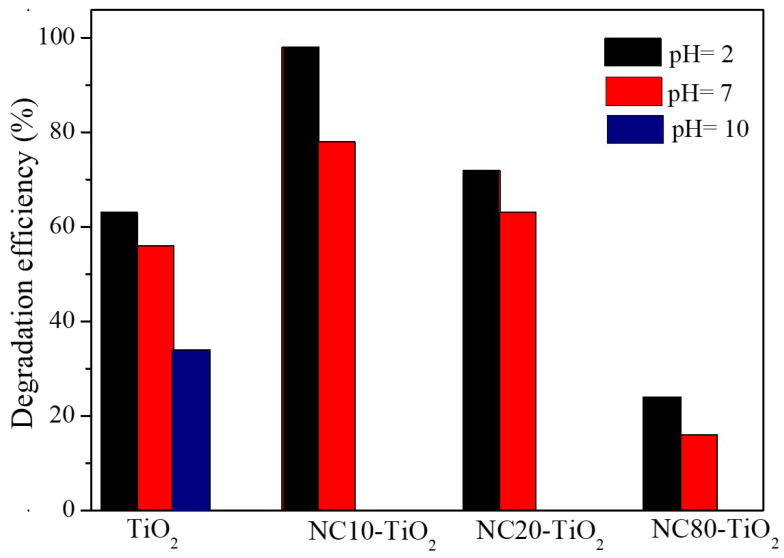
Percentages of the degradation efficiency for methyl orange obtained with different NC–TiO_2_ nanocomposites and TiO_2_ at different pH values.

It is well known that pH plays an important role in determining the efficiency of the photocatalytic degradation of sulfonated dyes, such as methyl orange, so we evaluated the activity of NC–TiO_2_ nanocomposites at different pHs, and the results are summarized in Figure 7. For all the nanocomposites, the best photocatalytic efficiency was obtained at pH = 2 under continuous UV irradiation. At basic pH, however, NC–TiO_2_ nanocomposites encountered intrinsic chemical instability, and it was not possible to carry out photocatalytic measurements. Such an issue was also reported by Comparelli et al. [40] for TiO_2_ nanoparticles covered by oleic acid, who assumed that such instability was owed to the removal of the oleic acid ligand from the surface of TiO_2_ nanoparticles caused by the enhanced solubility of the ligand at such an extreme pH. Moreover, the Raman characterization (Figure S2) performed on our NC–TiO_2_ nanocomposites after photocatalytic experiments at different pHs confirmed the hypothesis about the occurrence of some degradations at a basic pH. In particular, the Raman spectrum of NC20–TiO_2_ after photocatalysis at pH 10 showed no longer peaks typical of cellulose, while only Raman peaks of TiO_2_ anatase were visible.

Finally, the recyclability of the photocatalyst was evaluated for the NC10–TiO_2_ sample, being the most efficient in MO degradation. After each photocatalytic run, the sample was recovered from solution by centrifugation, washed with ethanol, and dried. The composite was then reused for a new MO photodegradation test at pH 2.

**Figure 8:**
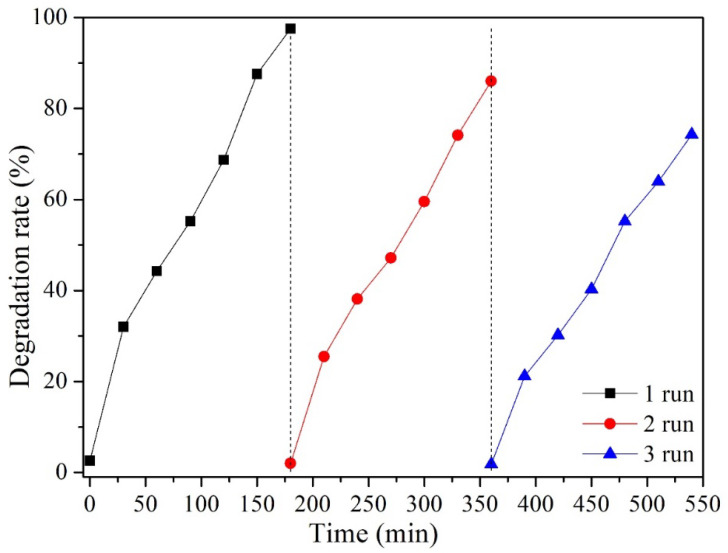
Evaluation of the degradation rate for methyl orange solution over UV irradiation NC10–TiO_2_ nanocomposites for three consecutive cycles.

The results were reported in Figure 8: after three times of repeated use, the degradation rate decreased from 98% to 75%, which could be attributed mainly to the inevitable loss of small amounts of photo-active material after every run (about 20% mass loss for NC10–TiO_2_ at the end of the collection processes).

**Figure 9:**
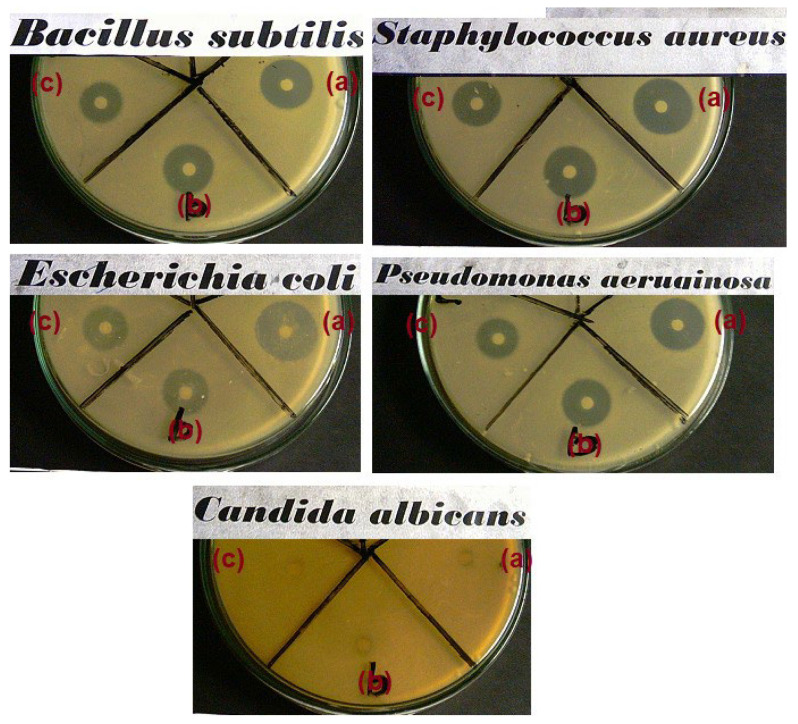
Antimicrobial activity for TiO_2_ (**a**), NC20–TiO_2_ (**b**), and NC80–TiO_2_ (**c**) nanocomposites.

The antimicrobial activity of NC–TiO_2_ nanocomposites was qualitatively assessed by applying an agar well diffusion disk test against four bacterial species, *Staphylococcus aureus, Bacillus subtilis* as Gram (+) bacteria, *Escherichia coli*, *Pseudomonas aeuroginosa* as Gram (–) bacteria, and a yeast pathogen *Candida albicans*. According to this method, the nanocomposites impregnated a paper filter placed on an agar plate. After that, the bacteria were allowed to grow on the agar plate and the evaluation of the diameter of the inhibitory zones allowed to determine the efficiency of bacterial growth inhibition [41]. The pure TiO_2_ and two nanocomposites having different NC contents (NC20–TiO_2_ and NC80–TiO_2_) were selected for this study. The antibacterial activity of the selected samples was represented in Figure 9.

**Figure 10:**
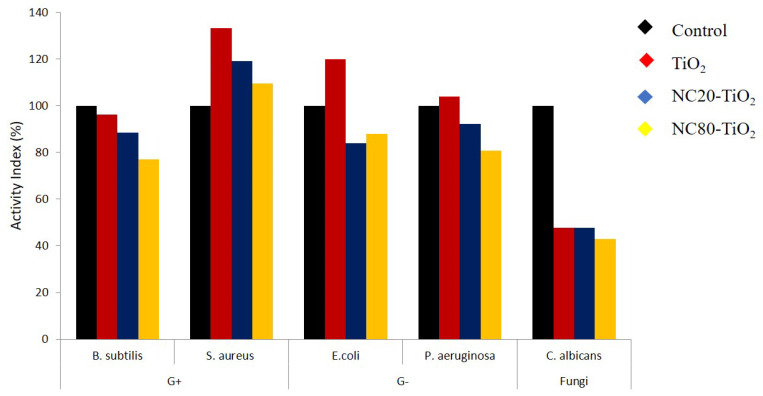
Antimicrobial activities (activity index %) for TiO_2_ and NC–TiO_2_ nanocomposites. Blank paper is added as control.

The results, expressed by measuring the size of the inhibition zone, were summarized in Table S2 and highlighted the influence of the different TiO_2_:NC ratios on the antimicrobial properties of the nanocomposites. Comparing the results, indeed, the formation of a clear inhibition zone around all the samples was evident, indicating that the NC–TiO_2_ nanocomposites possessed peculiar bactericide effects and were effective in inhibiting the growth of all the four bacteria species and *Candida albicans* fungi, as well as the neat TiO_2_. Furthermore, the results clearly indicated that the antimicrobial activity of the nanocomposites was induced by TiO_2_ nanoparticles, and it was strictly dependent on their concentration: the NC20–TiO_2_ nanocomposite sample showed much better antibacterial properties against *Bacillus subtilis* and *Staphylococcus aureus* than G(+) and *Pseudomonas aeruginosa* *as* G(*−*) bacteria. Our results were in accordance with those reported by Zong [31], which observed an improvement in the antibacterial properties of cellulose after its functionalization with TiO_2_ nanoparticles: in particular, when the contents of Ti increased, obvious inhibition zones in the Petri dishes were observed (0.4–2.8 mm, depending on Ti content). The exact mechanism involved in the inactivation of microorganisms by TiO_2_-based materials has not been clear until now, and it is usually associated with different phenomena involving, for example, the photocatalytic generation, under band-gap irradiation, of reactive oxygen species (ROS) with high oxidative potentials, oxidative stress caused by ROS, interruption of microorganisms’ DNA replication, and protein synthesis processes caused by reaction with ions released by nanoparticles [42]. Moreover, our results demonstrated that NC–TiO_2_ nanocomposites were more active against the Gram-positive *Staphylococcus aureus* and displayed very high activity compared to the standard drug (Figure 10). This behavior has already been observed [43,44] and could be explained considering the differences in the structure of the cell walls of these bacteria: Gram-negative bacteria (e.g., *Pseudomonas aeruginosa*) usually have an additional outer membrane consisting of lipopolysaccharide and peptidoglycan, which helps them to resist to the oxidative damage caused by the interaction with the TiO_2_ nanoparticles.

The good antimicrobial performance and the photocatalytic activity of the nanocellulose–TiO_2_ nanocomposites make these materials the key candidates in many important industrial sectors, such as food manufacturing and packaging, water remediation, pharmaceuticals, and medical treatments.

## 4. Conclusions

We demonstrated the successful one-pot synthetic procedure for the fabrication of nanocellulose–TiO_2_ nanocomposites. With regard to hydrothermal methods and other sol–gel synthesis methods that normally require high-temperature calcination steps or harmful solvents, the proposed approach is simple, environmentally friendly, low-cost, and energy-saving. Structural and morphological characterization revealed the formation of well crystalline anatase nanoparticles with a final size of about 5–6 nm. The chemical interactions between the surface chemical groups on the nanocellulose and the titania precursor are responsible for the significant narrowing of the energy band gap with respect to the pure TiO_2_, thus leading to a more efficient photocatalyst towards the photodegradation of methyl orange (almost 99% degradation after 180 min of UV illumination). In particular, the NC–TiO_2_ nanocomposites with a low amount of nanocellulose revealed particular efficiency in increasing the density of surface hydroxyl groups, which can easily react with holes to produce hydroxyl radicals that degrade dye molecules. Furthermore, the NC–TiO_2_ nanocomposites also possessed considerable antimicrobial activity against Gram-negative and Gram-positive bacterial strains. Therefore, this study provides new insight for fabrication of efficient NC–TiO_2_ photocatalysts and enables their application in different sectors, including water remediation and packaging.

## Figures and Tables

**Table 1:** Comparison of the photocatalytic efficiency NC–TiO_2_ with other reported works for the catalytic degradation of various organic pollutants.

Catalyst	Pollutant	Wavelength	Efficiency	Reference
TiO_2_/CNF	MO	365 nm	99% within 30 min	[20]
TiO_2_/NFC	MO	365 nm	95% within 60 min	[21]
CNF/TiO_2_	MB	UV	97% within 30 min	[30]
TiO_2_/RC	phenol	365 nm	96% within 360 min	[15]
TiO_2_/Agar	MB	UV	62% within 105 min	[25]
NC/TiO_2_	Mefenamic acid	UV	89% within 150 min	[39]
NC/TiO_2_	MO	365 nm	99% within 180 min	This work

## Data Availability

Data presented in this study are available on request from the authors.

## Supplementary Materials

The following supporting information can be downloaded at: https://www.mdpi.com/article/10.3390/ma15165789/s1, Figure S1: DTG analyses of NC–TiO_2_ nanocomposites; Table S1: TG, DTG, kinetic, and thermodynamic data from the thermal decomposition steps of prepared samples; Figure S2: Raman spectra of NC20–TiO_2_ sample after photocatalytic tests at different pH; Table S2: Diameter size of the inhibition zone for TiO_2_, NC20–TiO_2_, and NC80–TiO_2_ nanocomposites through different bacteria.

## References

[1] Saveleva M.S., Eftekhari K., Abalymov A., Douglas T.E.L., Volodkin D., Parakhonskiy B.V., Skirtach A.G. (2019). Hierarchy of Hybrid Materials—The Place of Inorganics-*in*-Organics in it, Their Composition and Applications. Front. Chem..

[2] Singh S., Chen H., Shahrokhi S., Wang L.P., Lin C.-H., Hu L., Guan X., Tricoli A., Xu Z.J., Wu T. (2020). Hybrid Organic−Inorganic Materials and Composites for Photoelectrochemical Water Splitting. ACS Energy Lett..

[3] Mir S.H., Nagahara L.A., Thundat T., Mokarian-Tabari P., Furukawa H., Khosla A. (2018). Organic-Inorganic Hybrid Functional Materials: An Integrated Platform for Applied Technologies. J. Electrochem. Soc..

[4] Adel A.M., Ibrahim A.A., El-Shafei A.M., Al-Shemy M.T. (2019). Inclusion complex of clove oil with chitosan/β-cyclodextrin citrate/oxidized nanocellulose biocomposite for active food packaging. Food Packag. Shelf Life.

[5] Adel A.M., Al-Shemy M.T., Diab M.A., El-Sakhawy M., Toro R.G., Montanari R., de Caro T., Caschera D. (2021). Fabrication of packaging paper sheets decorated with alginate/oxidized nanocellulose-silver nanoparticles bio-nanocomposite. Int. J. Biol. Macromol..

[6] Adel A.M., El-Shafei A.M., Ibrahim A.A., Al-Shemy M.T. (2019). Chitosan/Nanocrystalline Cellulose Biocomposites Based on Date Palm (*Phoenix dactylifera* L.) Sheath Fibers. J. Renew. Mater..

[7] Shopsowitz K.E., Stahl A., Hamad W.Y., MacLachlan M.J. (2012). Hard templating of nanocrystalline titanium dioxide with chiral nematic ordering. Angew. Chem..

[8] Zuo H.-F., Guo Y.-R., Li S.-J., Pan Q.-J. (2014). Application of microcrystalline cellulose to fabricate ZnO with enhanced photocatalytic activity. J. Alloy. Compd..

[9] Luo Y., Xu J., Huang J. (2014). Hierarchical nanofibrous anatase-titania-cellulose composite and its photocatalytic property. Cryst. Eng. Comm..

[10] Caschera D., Toro R.G., Federici F., Montanari R., de Caro T., Al-Shemy M.T., Adel A.M. (2020). Green approach for the fabrication of silver-oxidized cellulose nanocomposite with antibacterial properties. Cellulose.

[11] Toro R.G., Diab M., de Caro T., Al-Shemy M., Adel A., Caschera D. (2020). Study of the Effect of Titanium Dioxide Hydrosol on the Photocatalytic and Mechanical Properties of Paper Sheets. Materials.

[12] Malviya A., Jaspal D., Sharma P., Dubey A. (2015). Isothermal mathematical modeling for decolorizing water—A comparative approach. Sustain. Environ. Res..

[13] Nyangiwe N.N., Baatjie B., Greyling C., Khenfouch M., Maaza M. (2018). The decolourisation of Methyl Orange and textile effluent under UV using commercial and synthesized nano-TiO_2_. J. Phys. Conf. Ser..

[14] Anaya-Esparza L.M., Ruvalcaba-Gómez J.M., Maytorena-Verdugo C.I., González-Silva N., Romero-Toledo R., Aguilera-Aguirre S., Pérez-Larios A., Montalvo-González E. (2020). Chitosan-TiO_2_: A Versatile Hybrid Composite. Materials.

[15] Mohamed M.A., Salleh W.N.W., Jaafar J., Ismail A.F., Mutalib M.A., Sani N.A.A., Asri S., Ong C.S. (2016). Physiochemical characteristic of regenerated cellulose/N-doped TiO_2_ nanocomposite membrane fabricated from recycled newspaper with photocatalytic activity under UV and visible light irradiation. Chem Engin. J..

[16] Zhan C., Li Y., Sharma P.R., He H., Sharma S.K., Wang R., Hsiao B.S. (2019). A study of TiO_2_ nanocrystal growth and environmental remediation capability of TiO_2_/CNC nanocomposites. RSC Adv..

[17] Zhao X., Zhang G., Zhang Z. (2020). TiO_2_-based catalysts for photocatalytic reduction of aqueous oxyanions: State-of-the-art and future prospects. Environ. Int..

[18] Li G., Park S., Rittmann B.E. (2012). Developing an efficient TiO_2_-coated biofilm carrier for intimate coupling of photocatalysis and biodegradation. Water Res..

[19] Toro R.G., Calandra P., Federici F., de Caro T., Mezzi A., Cortese B., Pellegrino A.L., Malandrino G., Caschera D. (2020). Development of superhydrophobic, self-cleaning, and flame-resistant DLC/TiO_2_ melamine sponge for application in oil–water separation. J. Mater. Sci..

[20] Liu G.-Q., Pan X.-J., Li J., Li C., Ji C.-L. (2021). Facile preparation and characterization of anatase TiO_2_/nanocellulose composite for photocatalytic degradation of methyl orange. J. Saudi Chem. Soc..

[21] Xiao H., Li J., He B. (2017). Anatase-Titania Templated by Nanofibrillated Celluloseand Photocatalytic Degradation for Methyl Orange. J. Inorg. Organomet Polym..

[22] Adel M.A., El-Gendy A.A., Mohamed D.A., Abou-Zeid R.E., El-Zawawy W.K., Dufresne A. (2016). Microfibrillated cellulose from agricultural residues. Part I: Papermaking application. Ind. Crops Prod..

[23] Adel A.M., El-Shafei A., Ibrahim A., Al-Shemy M.T. (2018). Extraction of oxidized nanocellulose from date palm (*Phoenix dactylifera* L.) sheath fibers: Influence of CI and CII polymorphs on the properties of chitosan/bionanocomposite films. Ind. Crops Prod..

[24] Ibrahim A.A., Adel A.M., AbdEl–Wahab Z.H., Al–Shemy M.T. (2011). Utilization of carboxymethyl cellulose based on bean hulls as chelating agent. Synthesis, characterization and biological activity. Carbohydr. Polym..

[25] Najafidoust A., Allahyari S., Rahemi N., Tasbihi M. (2020). Uniform coating of TiO_2_ nanoparticles using biotemplates for photocatalytic wastewater treatment. Ceram. Int..

[26] Schenzel K., Fischer S. (2001). NIR FT Raman Spectroscopy—A Rapid Analytical Tool for Detecting the Transformation of Cellulose Polymorphs. Cellulose.

[27] Schenzel K., Almlof H., Germgard U. (2009). Quantitative analysis of the transformation process of cellulose I→cellulose II using NIR FT Raman spectroscopy and chemometric methods. Cellulose.

[28] Adel A.M., El-Wahab Z.H.A., Ibrahim A.A., Al-Shemy M.T. (2010). Characterization of microcrystalline cellulose prepared from lignocellulosic materials. Part I: Acid catalyzed hydrolysis. Bioresour. Technol..

[29] Shankar S., Rhim J.W. (2016). Preparation of nanocellulose from micro-crystalline cellulose: The effect on the performance and properties of agar-based composite films. Carbohydr. Polym..

[30] Li S., Hao X., Dai X., Tao T. (2018). Rapid Photocatalytic Degradation of Pollutant from Water under UV and Sunlight via Cellulose Nanofiber Aerogel Wrapped by TiO_2_. J. Nanomater..

[31] Zong E., Wang C., Yang J., Zhu H., Jiang S., Liu X., Song P. (2021). Preparation of TiO_2_/cellulose nanocomposites as antibacterial bio-adsorbents for effective phosphate removal from aqueous medium. Int. J. Biolog. Macromol..

[32] De Almeida J.C., Correâ M.T., Koga R.H., Del Duque D.M.S., Lopes O.F., Gelson T.S., da Silva T., Ribeiro C., de Mendonça V.R. (2020). Crystallization time in ZnO: The role of surface OH groups in its photoactivity. New J. Chem..

[33] Cao W., Li J., Martí-Rossellò T., Zhang X. (2019). Experimental study on the ignition characteristics of cellulose, hemicellulose, lignin and their mixtures. J. Energy Inst..

[34] Ceylan Ö., Van Landuyt L., Rahier H., De Clerck K. (2013). The effect of water immersion on the thermal degradation of cotton fibers. Cellulose.

[35] Coats A.W., Redfern J.P. (1964). Kinetic Parameters from Thermogravimetric Data. Nature.

[36] El-Sabour M.A., Mohamed A.L., El-Meligy M.G., Al-Shemy M.T. (2021). Characterization of recycled waste papers treated with starch/organophosphorus-silane biocomposite flame retardant. Nord. Pulp Pap. Res. J..

[37] Li Y., Zhang J., Zhan C., Kong F., Li W., Yang C., Hsiao B.S. (2020). Facile synthesis of TiO_2_/CNC nanocomposites for enhanced Cr(VI) photoreduction: Synergistic roles of cellulose nanocrystals. Carbohydr. Polym..

[38] Petrella A., Spasiano D., Cosma P., Rizzi V., Race M., Mascolo M.C., Ranieri E. (2021). Methyl Orange Photo-Degradation by TiO_2_ in a Pilot Unit under Different Chemical, Physical, and Hydraulic Conditions. Processes.

[39] Rathod M., Moradeeya P.G., Haldar S., Basha S. (2018). Nanocellulose/TiO_2_ composites: Preparation, characterization and application in the photocatalytic degradation of a potential endocrine disruptor, mefenamic acid, in aqueous media. Photochem. Photobiol. Sci..

[40] Comparelli R., Fanizza E., Curri M.L., Cozzoli P.D., Mascolo G., Passino R., Agostiano A. (2005). Photocatalytic degradation of azo dyes by organic-capped anatase TiO_2_ nanocrystals immobilized onto substrates. Appl. Catal. B Envir..

[41] Matar M.J., Ostrosky-Zeichner L., Paetznick V.L., Rodriguez J.R., Chen E., Rex J.H. (2003). Correlation between E-test, disk diffusion, and microdilution methods for antifungal susceptibility testing of fluconazole and voriconazole. Antimicrob. Agents Chemother..

[42] Bhuiyan M.S.H., Miah M.Y., Pail S.C., Aka T.D., Saha O., Rahaman M.M., Sharif M.J.I., Habiba O., Ashaduzzaman M. (2020). Green synthesis of iron oxide nanoparticle using Carica papaya leaf extract: Application for photocatalytic degradation of Remazol yellow RR dye and antibacterial activity. Helyon.

[43] Slavin Y.N., Asnis J., Hafeli U.O., Bach H. (2017). Metal nanoparticles; understanding the mechanism behind antibacterial activity. Int. J. Nanobiotech..

[44] Maslana K., Zywicka A., Wenelska K., Mijowska E. (2021). Boosting of Antibacterial Performance of Cellulose Based Paper Sheet via TiO_2_ nanoparticles. Int. J. Molec. Sci..

