# Supplementary material for: A Facile One-Pot Approach to the Fabrication of Nanocellulose–Titanium Dioxide Nanocomposites with Promising Photocatalytic and Antimicrobial Activity
Source: Materials (Basel). 2022 Aug 22;15(16):5789. doi: 10.3390/ma15165789 (PMC9415876; doi:10.3390/ma15165789)
Supplement: Supplementary file 1 [file materials-15-05789-s001.zip › materials-1861181-supplementary.pdf]

A facile one-pot approach to the fabrication of Nanocellulose/Titanium Dioxide nanocomposites with promising photocatalytic and antimicrobial activity

Roberta G. Toro[1,*], Abeer M. Adel[2], Tilde de Caro[1], Bruno Brunetti[3], Mona T. Al-Shemy[2], Daniela Caschera[1]

[1] Institute for the Study of Nanostructured Materials, ISMN-CNR, Via Salaria km 29,300 Monterotondo, Rome, Italy
[2] National Research Centre, Cellulose and Paper Department, 33El-Bohouth St. (Former El-Tahrir St.), Dokki, P.O. 12622, Giza, Egypt
[3] Institute for the Study of Nanostructured Materials, ISMN-CNR, c/o Dipartimento di Chimica, Università degli Studi di Roma "La Sapienza", Piazzale Aldo Moro 5, Rome, Italy

*Corresponding Author

Roberta G. Toro

e-mail: robertagrazia.toro@cnr.it

Phone: +390690672828

Orcid: https://orcid.org/0000-0002-8720-4086

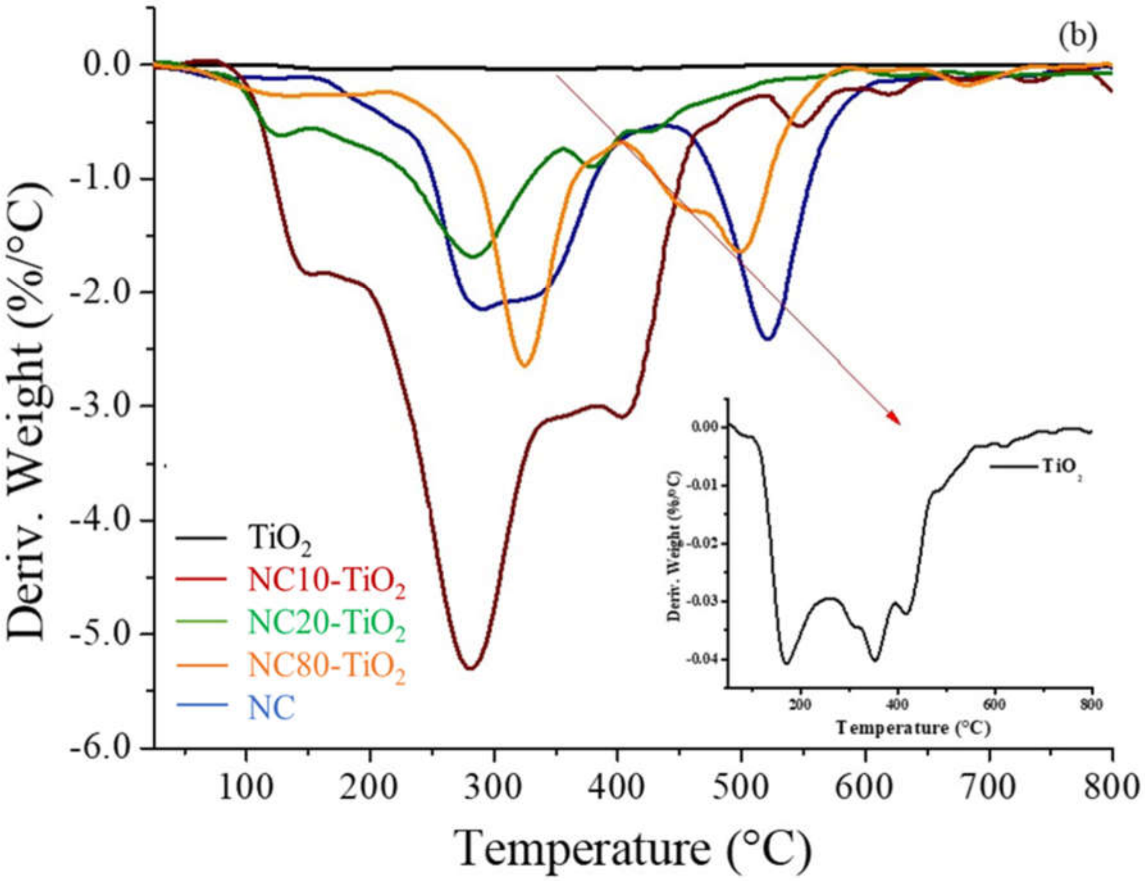

Figure S1: DTG analyses for (–) TiO_2, (–) NC, (–) NC10-TiO_2, (–) NC20-TiO_2, and (–) NC80-TiO_2.

Sample	Step	T_m (°C)	Wt_{loss} (%)	RC_{650} (%)	A (s^{-1})	Ea (kJ mol^{-1})	ΔH (kJ mol^{-1})	ΔS (JK^{-1} mol^{-1})	ΔGa (kJ mol^{-1})	r^2
TiO_2	I<br>II	170<br>354	2.22<br>4.34	93.45	96.89×10^5<br>117.22	73.65<br>47.89<br>∑121.54	69.97<br>42.68<br>∑112.64	-0.115<br>-0.212<br>∑-0.326	120.68<br>175.31<br>∑295.99	0.93<br>0.93
NC10-TiO_2	I	281	16.8	83.49	5.98	29.00<br>∑29.00	24.40<br>∑24.40	-0.235<br>∑-0.235	154.73<br>∑154.73	0.98
N20-TiO_2	I	283	23.4	76.19	3.36	26.05<br>∑26.05	21.43<br>∑21.43	-0.240<br>∑-0.240	154.91<br>∑154.91	0.95
NC80-TiO_2	I<br>II	325<br>499	39.3<br>32.7	21.28	82.57×10^4<br>40.09×10^6	84.25<br>130.68<br>∑214.93	79.28<br>124.26<br>∑203.54	-0.138<br>-0.107<br>∑-0.245	161.48<br>207.09<br>∑368.58	0.98<br>0.98
NC	I<br>II	290<br>522	50.93<br>33.77	9.67	18.58×10^2<br>1.48×10^8	54.84<br>146.12<br>∑200.96	50.16<br>139.51<br>∑189.67	-0.188<br>-0.097<br>∑-0.284	155.81<br>216.39<br>∑372.19	0.99<br>0.98

Table S1: TG, DTG, kinetic and thermodynamic data from the thermal decomposition steps of prepared samples

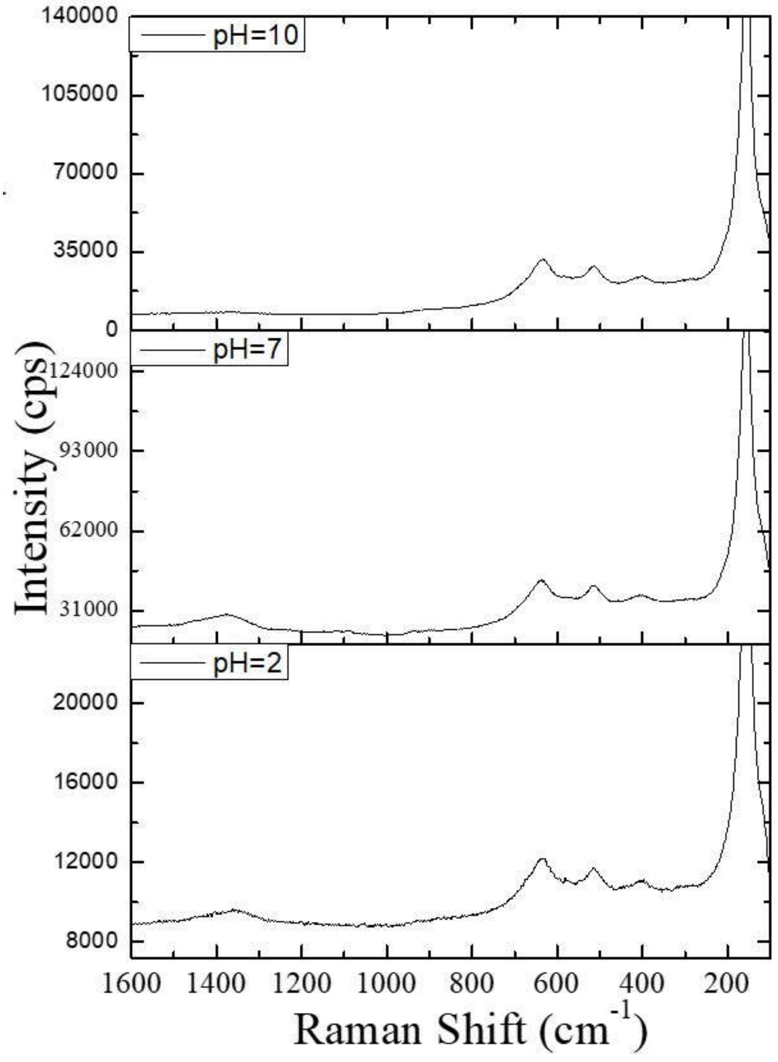

Figure S2: Raman spectra of NC20-TiO_2 sample after photocatalytic tests at different pH

<table>
<tr><th colspan="2" rowspan="4">Sample</th><th colspan="5">Inhibition zone diameter (mm/Sample)</th></tr>
<tr><th colspan="4">Bacterial species</th><th>Fungi</th></tr>
<tr><th colspan="2">G^+</th><th colspan="2">G^-</th><th rowspan="2">Candida albicans</th></tr>
<tr><th>Bacillus subtilis</th><th>Staphylococcus aureus</th><th>Escherichia coli</th><th>Pseudomonas aeruginosa</th></tr>
<tr><td rowspan="2">Standard</td><td>Ampicillin Antibacterial agent</td><td>26</td><td>21</td><td>25</td><td>26</td><td>--</td></tr>
<tr><td>Amphotericin B Antifungal agent</td><td>--</td><td>--</td><td>--</td><td>--</td><td>21</td></tr>
<tr><td colspan="2">TiO_2</td><td>25</td><td>28</td><td>30</td><td>27</td><td>10</td></tr>
<tr><td colspan="2">NC20-TiO_2</td><td>23</td><td>25</td><td>21</td><td>24</td><td>10</td></tr>
<tr><td colspan="2">NC80-TiO_2</td><td>20</td><td>23</td><td>22</td><td>21</td><td>9</td></tr>
</table>

Table S2: Diameter size of the inhibition zone for TiO_2, NC20-TiO_2, and NC80-TiO_2 nanocomposites through different bacteria.
